# Oviposition and father presence reduce clutch cannibalism by female poison frogs

**DOI:** 10.1186/s12983-019-0304-2

**Published:** 2019-03-22

**Authors:** Sandra Spring, Marion Lehner, Ludwig Huber, Eva Ringler

**Affiliations:** 1 Department of Integrative Zoology, University of Vienna, Althanstrasse 14, A-1090 Vienna, Austria; 2 Messerli Research Institute, University of Veterinary Medicine Vienna, Medical University Vienna, University of Vienna, Veterinaerplatz 1, A-1210 Vienna, Austria

**Keywords:** Cannibalism, Parental care, Territoriality, Dendrobatidae, *Allobates femoralis*

## Abstract

**Background:**

The consumption of conspecific young by adult individuals is a common phenomenon across various animal taxa. Possible adaptive benefits of such behaviour include the acquisition of nutrients, decreased competition for one’s own offspring, and/or increased mating opportunities. Clutch cannibalism has occasionally been observed in several species of Neotropical poison frogs, but the circumstances under which this behaviour occurs has rarely been investigated experimentally. Recent experiments with the poison frog *Allobates femoralis* have shown that males indiscriminately transport all clutches located inside their own territory to bodies of water, but become highly cannibalistic when taking over a new territory. Females are able to indirectly discriminate between their own and foreign clutches by location and take over transport duties of their own clutches only in the absence of the father. Cannibalism by *A. femoralis* females has not been previously observed. We thus asked if, and under which circumstances, cannibalism of unrelated clutches by female *A. femoralis* would occur, by manipulating the presence of the clutch’s father, the female’s own reproductive state, and the female’s familiarity with the environment.

**Results:**

Females clearly cannibalize foreign clutches. Cannibalism was most pronounced when the female had not recently produced her own clutch and the father of the foreign clutch was absent. The female’s familiarity with the area had no significant influence on the likelihood of cannibalism to occur.

**Conclusions:**

Our data indicate that both previous oviposition and the father’s presence reduce cannibalistic behaviour in *A. femoralis* females. Cannibalistic females may gain nutritional benefits or enhanced inclusive fitness by preying on other females’ offspring. The finding that the father’s presence at the clutch site/territory was sufficient to reduce cannibalism by females suggests a prominent role of male territoriality for the evolution of male parental care.

**Electronic supplementary material:**

The online version of this article (10.1186/s12983-019-0304-2) contains supplementary material, which is available to authorized users.

## Background

Cannibalism, the consumption of conspecific individuals, is a common phenomenon in various animal taxa [1]. It may occur between individuals of the same life history cohort, for example between adults (e.g. several arachnids and insects; [2, 3]) or between juvenile individuals often in the context of sibling competition (e.g. amphibian larvae, [4–6]). Cannibalism also occurs between different life history stages; for example, if adult individuals consume conspecific young [7–9]. While at first glance such destructive behaviour towards conspecifics may seem maladaptive from an evolutionary perspective, several factors have been identified that may explain how such behaviour could become adaptive [8]. Cannibalistic individuals benefit by gaining nutrients [9, 10], particularly when other food sources are scarce [11, 12]. Destructive behaviour towards other individuals’ offspring, which does not necessarily include the consumption thereof, may also serve to reduce the amount of intraspecific competition experienced by the perpetrator’s own offspring [10, 13]. For example, in meerkats pregnant dominant females often kill pups of other subordinate females to increase the helper-to-pup-ratio for their own offspring [14, 15]. Killing unrelated conspecific offspring is also known as a behavioural strategy to obtain earlier access to potential mating partners [1, 16]. For example, male lions kill cubs, causing mothers to become reproductively receptive sooner [17].

Two types of cannibalism of conspecific young by adults can be distinguished: heterocannibalism (cannibalizing genetically unrelated individuals; [18]) and filial cannibalism (cannibalizing one’s own offspring; [19]). One potential risk of adults killing conspecific young is the accidental killing of one’s own young, or at least closely related individuals [1] which may have considerable impact on one’s own inclusive fitness [20, 21]. Consequentially, in several species parents may either directly [6] and/or indirectly [22, 23] discriminate between their own offspring and those of others. For example, parental male bluegill sunfish discriminate between kin and non-kin nestlings based both the visual presence of cuckolder males during spawning, and olfactory cues released by newly hatched eggs [24, 25]. In two species of predaceous mites, *Phytoseiulus macropilis* and *P. persimilis*, females have been shown to recognize and avoid cannibalizing related larvae, but the exact mechanism underlying this discriminative behaviour is not known [7].

In amphibians, cannibalism occurs between and within different life-cycle stages (egg-larva-metamorph-adult), and both hetero- and filial cannibalism have been documented [26]. In species where adults and conspecific larvae inhabit the same habitat, adults commonly feed on small prey, including conspecific eggs and larvae [27–29]. Filial clutch cannibalism is known from several fish, where selective cannibalism also has been shown to enhance egg survival [8]. In amphibians, similar behaviour is mainly reported from plethodontid salamanders during parental care (e.g. *Desmognathus fuscus fuscus*; [30]; *Plethodon cinereus*; [22]; *Desmognathus ochrophaeus* [31, 32]), where the females consume dead or infected eggs to protect the remaining clutch from being infected. Tactical egg cannibalism by conspecifics has been shown to constitute a major source of egg mortality in a wide range of amphibian species (e.g. *Taricha torosa;* [33], *Eleutherodactylus coqui* [34, 35]), however the underlying motivations and behavioural strategies are poorly understood. One of the evolutionary benefits of parental care is that it increases offspring survival by minimizing predation, including predation by conspecifics [36–38]. Animal species that feature both parental care and cannibalism thus provide ideal models for studying the adaptive significance of parenting with respect to predation threat by conspecifics.

Dendrobatid frogs display elaborate reproductive behaviour, including parental care [39–41]. Almost all dendrobatid frogs deposit their eggs on land, which is considered an evolutionary adaptation to high predation pressure on eggs in water, but which in turn may have facilitated predation by terrestrial egg predators, including conspecifics [39, 42, 43]. Egg cannibalism by conspecifics has been occasionally observed in many poison frog species, with most cases reported in captive females [44, 45], but also in the field [46]. However, this behaviour has rarely been studied using manipulative experimental approaches, and consequentially the underlying circumstances that either promote or inhibit clutch cannibalism by adult frogs remain poorly understood [47]. Examples of clutch cannibalism in dendrobatid frogs include *Dendrobates auratus*, where males are highly polygynous and females compete for males and may cannibalize eggs of other females, which has been suggested as a behavioural mechanism to gain a monopoly on the male’s parental care [45, 46, 48]. Adult *Oophaga arborea* females were found to cannibalize unrelated clutches, but the reasons for cannibalism in this species remain unclear [44]. In one pair of *Epipedobates tricolor* in captivity the female cannibalized her own clutch if the father was removed from the tank a few days after mating, but cared for the clutch if the father was removed directly after mating [49]. Clutch cannibalism has also been, to a lesser extent, reported in male dendrobatid frogs. For example, anecdotal reports of male *Oophaga pumilio* consuming unrelated egg suggest that cannibalism might speed up female reproductive receptiveness because parenting females are unlikely to mate [50]. In *Colostethus palmatus* males usually care for a single clutch at a time and do not leave the clutch site during brooding. However, if the clutch is manipulated experimentally, e.g. covered or removed to another site, or the male was prevented from visiting the clutch for 2 days, he stops tending to his eggs and sometimes even cannibalizes them [51], possibly because he no longer recognizes them as his own. Selective clutch cannibalism has recently been reported in male *Allobates femoralis,* a dendrobatid frog that occurs in lowland forests of the Amazon basin and Guiana Shield [52]. Although males indiscriminately transport any clutch, including unrelated ones, that is located inside their territory [23], they become highly cannibalistic when taking over a new territory [53]. They remove the former territory holder’s offspring and thereby minimize risks and costs of misdirected parental care [53].

Although clutch cannibalism in poison frogs has mainly been reported for females, in *A. femoralis* females have never been observed to prey on clutches – neither in the field nor in the lab. However, females have been shown to be able to distinguish between their own and unrelated clutches by exact location when taking over tadpole transport after experimental father removal [23], which would provide ideal prerequisites for selective cannibalism on non-related clutches to evolve. We thus asked if *A. femoralis* females become cannibalistic when confronted with unrelated offspring. To answer this question, we designed an experiment where we tested different conditions that represent biologically relevant situations in which females might show cannibalistic behaviour. Given potential differences in costs and benefits of clutch cannibalism, we hypothesized that females may become cannibalistic particularly when confronted with unrelated clutches that are (1) outside their home area (2) not guarded by the respective father and/or (3) when they have not laid their own clutch.

## Material and methods

### Study species

Throughout the reproductive season, male *A. femoralis* are highly territorial and actively defend their territories against calling intruders [54, 55]. Females show site fidelity, but do not actively defend the area [56, 57]. *Allobates femoralis* has a polygamous mating system, where males and females have multiple mating partners [58]. Mating occurs inside the male’s territory after a long and complex courtship [59] (Stückler S, unpublished data), and egg clutches are deposited in the leaf litter. Females leave the male territory soon after mating [57, 60]. About 3 weeks after oviposition the tadpoles hatch and are transported by the father to small pools of water, where larval development is completed [61, 62]. Females only transport tadpoles if the male is absent at the time when tadpole transport is due [63], in which case, they will only select their own clutch for transport based on its exact location [23].

### Setup/housing

The experiments were conducted from 10 February to 21 December 2016 in the animal care facilities of the Biocenter Althanstrasse of the University of Vienna. Each experiment was performed in a standard glass terrarium with a floor area of 60 × 40 cm and 40 cm height. Each terrarium had the same furnishing and equipment; the side walls and back wall were covered with xaxim and cork mats, the floor was covered with expanded clay pebbles. Dried oak leaves were provided as a substrate for egg clutches. Each terrarium contained a glass bowl of 12 cm diameter filled with approximately 350 ml of reverse osmosis water, a small plant, half a coconut shell as a hiding place, and a branch. The front of each terrarium was covered with fabric to prevent visual contact with other individuals and other disturbances. Climatic conditions were similar to natural conditions in French Guiana and standardized in all terraria through an automatic heating, lighting, and raining system. The temperature ranged from 19 °C at night to 30 °C during the day. Lights were on from 7 a.m. to 7 p.m. and humidity was constantly at 100%. All frogs are fed every second day with wingless fruit flies.

### Experimental design

**Fig. 1:**
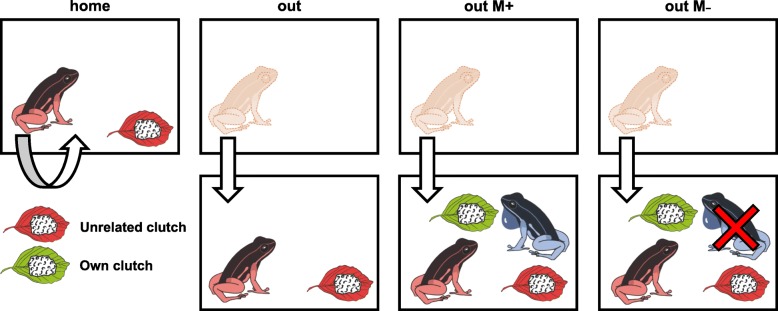
Experimental design. “home”: an unrelated clutch (red) was placed inside a female’s home terrarium. “out”: females were transferred to an unfamiliar empty terrarium that contained an unrelated clutch. “out M+”: females were transferred to a new terrarium with an unrelated clutch and a guarding father. In this condition, females usually produced immediately an own clutch (green) with the male. “out M-”: same as in the previous condition, except that the male was removed after clutch production with the test female

We pseudo randomly assigned females (total *N* = 40 females) to one of the four test conditions (*N* = 10 females/condition). No female participated twice in any trial. In all trials we placed an unrelated clutch inside the experimental terrarium, but manipulated the presence of the father, the presence of the tested female’s own clutch, and the familiarity of the female with the given area (Fig. 1). In the condition “home” we tested if females prey on unrelated clutches when encountered inside their home terrarium. For this, females were shortly (5–10 min) removed from their terrarium, an unrelated clutch (i.e. a clutch from another breeding pair) was placed inside the tank, and subsequently the female was returned to her home terrarium. In “out” we tested if females cannibalize unrelated clutches in an unoccupied and unfamiliar location. Here, females were transferred to an unfamiliar empty terrarium that only contained an unrelated clutch. In “out M+” we tested if females cannibalize unrelated clutches in a foreign terrarium where the respective father was present. As after the first trials of “out M+”, we recorded that females and males immediately started courting and in most cases produced a novel clutch, we added another condition to better disentangle the effects of oviposition and male presence. In “out M-”, females were transferred to a foreign terrarium with an unrelated clutch and the respective father present (like in “out M+”); however, the male was removed from the terrarium immediately after clutch production.

Females were caught from their terraria with transparent plastic bags and then returned or transferred to the same or a new terrarium, depending on the condition, to assure equal handling effects across all trials. There was no significant difference in either the developmental stage, measured in days after oviposition (Kruskal-Wallis test; *χ*^2^ = 0.296, df = 3, *p* = 0.961), or in the number of embryos per clutch (Kruskal-Wallis test; *χ*^2^ = 0.496, df = 3, *p* = 0.920) in the clutches used for experimentation across all conditions. All experiments were filmed by using digital full-HD video surveillance cameras (IndigoVision, BX400 HD Minidome) that were installed on top of the terraria. Cameras filmed from 7 a.m. to 7 p.m., corresponding to the duration of lighting in the room. The zoom and focus were adjusted so that the whole clutch and surrounding area were fully and clearly visible. Every other day the clutches were additionally inspected directly in the terraria and embryos were counted to verify the count from camera recordings. The videos were visually inspected via the computer program IndigoVision Control Center and the following behaviours and parameters were transcribed: the occurrence of cannibalism in a given trial (yes/no), the number of cannibalistic events (c_events), and the sum of consumed embryos (sum_embryos) per female. Additionally, we noted the degree of cannibalism (absent/partial/full clutch cannibalism), the percentage of the clutch that was cannibalized per female, and possible tadpole transport. The dataset is provided in the Additional file 2: Dataset S1.

### Analysis

All analyses were performed in the R Studio environment [64]. Shapiro-Wilk tests were used to test for normality of data, and non-parametric tests and measures are provided where data significantly deviated from normal distribution. Significant differences across conditions regarding the occurrence of cannibalism (yes/no) was tested with the Fisher’s exact test. We used Kruskal-Wallis tests to investigate significant differences across conditions regarding the number of cannibalistic events and the sum of embryos consumed. If omnibus tests showed statistically significant differences across groups, pairwise post-hoc analyses were performed for which the *p*-values were adjusted using false-discovery-rate (fdr) corrections to reduce the Type 1 error rate [65]. For the pairwise comparisons of the Fisher’s exact test (hereafter “Pairwise Fisher’s exact test”) we used the function “pairwiseNominalIndependence” from the package “rcompanion” [66]. The pairwise comparisons of the Kruskal-Wallis test were conducted with the “pairwise.wilcox.test” function.

## Results

**Fig. 2:**
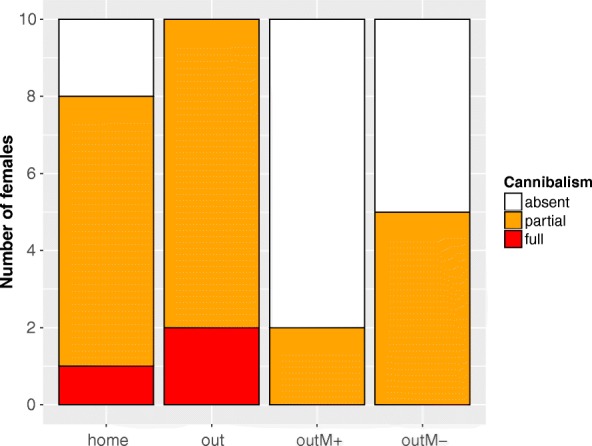
Degree of cannibalism across all four test conditions. Absent (white), partial (orange), full (red) clutch cannibalism


We observed clutch cannibalism in female *A. femoralis* across all experimental conditions (25 out of 40 trials, 62,5%, see also Additional file 1: Movie S1). Cannibalistic behaviour of females however was lower when fathers were present and after oviposition (“out M+”: *N* = 2 out of 10 females, “out M-”: *N* = 5 out of 10 females; Fig. 2). Females never transported any of the tadpoles from the unrelated clutches in any condition (*N* = 0 out of 40 trials).

**Additional file 1: Movie S1.** Clutch cannibalism by an *A. femoralis* female. (MP4 21039 kb)


We found significant differences across conditions for all tested parameters, namely the overall occurrence of cannibalism (Fisher’s exact test; *p* = 0.001), the number of cannibalistic events (Kruskal-Wallis test*; χ*^*2*^ = 13.60, df = 3, *p* = 0.003), and the sum of consumed embryos (Kruskal-Wallis test; *χ*^2^ = 14.47, df = 3, *p* = 0.002).

Cannibalism occurred more frequently in the two conditions completely without a male (8 of 10 females in “home” and 10 of 10 females in “out”). Also complete clutch cannibalism was only present in the two conditions where no male was present at any time during the experiment (Fig. 2). The occurrence of cannibalism was most likely to occur if females were placed in a new terrarium without a male (10 of 10 females in “out”), and lowest when a male was present throughout the experiment (2 of 10 females in “out M+”, Fig. 2). The two cannibalistic individuals in “out M+” were the only two in this condition that did not produce their own clutch during the course of the experiment. Half of the females cannibalized the unrelated clutch when they were in a new terrarium with a male that was removed after they produced their own clutch together (5 of 10 females in “out M-”, Fig. 2). None of the females preyed on own clutches.

**Fig. 3:**
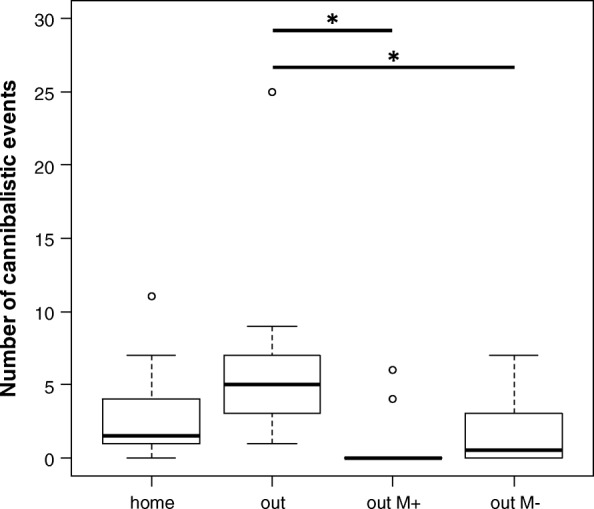
Number of cannibalistic events. Boxplots showing the number of cannibalistic events by all tested females across the four test conditions. Lines and asterisks indicate statistically significant differences between groups (*p* < 0.05, corrected for multiple comparisons)

**Fig. 4:**
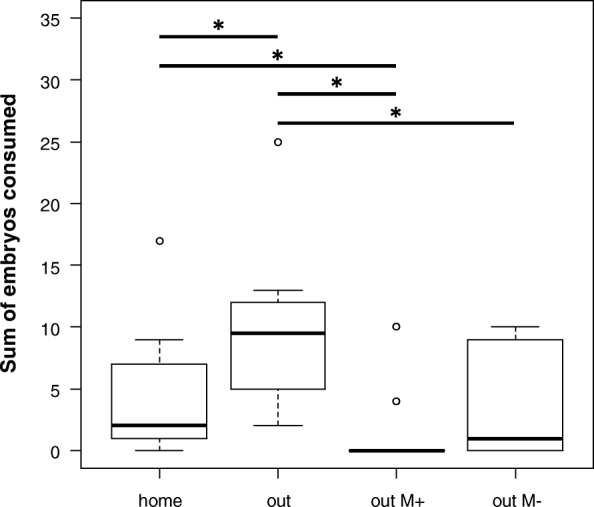
Sum of embryos consumed by females. Boxplots showing the sum of embryos that were consumed by tested females across the four test conditions. Lines and asterisks indicate statistically significant differences between groups (*p* < 0.05, corrected for multiple comparisons)

Pairwise comparisons revealed that the biggest differences in all parameters tested were found between the condition where females were outside their home tank and without any male (“out”) and the condition where they were moved to a foreign tank with a male present throughout (“out M+”, Table 1). In unfamiliar surroundings in the absence of a guarding male, significantly more females cannibalized clutches (Pairwise Fisher’s exact test, *p* = 0.004; Fig. 2), conducted more cannibalistic events (Wilcoxon Rank Sum test, *p* = 0.011; Fig. 3), and consumed higher numbers of embryos (Wilcoxon Rank Sum test, *p* = 0.007; Fig. 4), than when the father was present. We also found significant differences in the number of cannibalistic events (Wilcoxon Rank Sum test, *p* = 0.035), and the sum of embryos consumed by females (Wilcoxon Rank Sum test, *p* = 0.031) between conditions “out” and “out M-”. When females were in their home terrarium (“home”) the sum of cannibalized embryos was lower (Wilcoxon Rank Sum test, *p* = 0.05) than in trials where females were placed in a new terrarium without a male (“out”). Also when females were in a new terrarium with their own clutch and the father was present (“out M+”) the sum of consumed embryos was lower (Wilcoxon Rank Sum test, *p* = 0.050) than when females were in their home terrarium without a male or their own clutch (“home”). There was a trend that more females were cannibalistic (Pairwise Fisher’s exact test, *p* = 0.065) and showed cannibalistic behaviour at higher rates (c_ events, Wilcoxon Rank Sum test, *p* = 0.071) in the “home” condition versus “out M+”. All other pairwise comparisons revealed no significant differences (Table 1).

**Table 1:** Pairwise post-hoc tests between all test conditions

	cannibalism	c_events	sum_embryos
home vs. out	0.474	0.093	0.050*
home vs. out M+	0.065	0.071	0.050*
home vs. out M-	0.420	0.332	0.509
out vs. out M+	0.004*	0.011*	0.007*
out vs. out M-	0.065	0.035*	0.031*
out M- vs. out M+	0.420	0.321	0.298

cannibalism: occurrence of cannibalism; c_events: number of cannibalistic events per female; sum_embryos: sum of consumed embryos per female. *P*-values lower (or equal) than 0.05 are indicated with “*”

## Discussion

In this study we show selective clutch cannibalism in female *Allobates femoralis* in response to different spatial and reproductive conditions. Cannibalism was lower when fathers were present and after oviposition by the tested female. In the following we discuss possible reasons and/or motivations for this behaviour.

### Nutritional motivation

We suggest that one of the reasons for a female to cannibalize foreign clutches is to obtain valuable nutrients for producing her own clutches, as amphibian eggs are rich in phosphorus and calcium [26]. In many anurans conspecific eggs are an important part of an adults’ diet (e.g. *Xenopus laevis* [28], *Eleutherodactylus coqui* [34, 35], *Rana ridibunda* [67]). However, nutritional motivation alone cannot explain the observed differences across experimental conditions. All females in our study received equal and sufficient amounts of food over the course of the experiment, thus no differences in food supply existed between the test conditions. *Allobates femoralis* females also never cannibalized their own clutches in our study. Although nutritional motivation may be a promotor for clutch cannibalism in our experiment, the differences between the conditions suggest a different factor to be the main reason for female cannibalism in *A. femoralis*.

### Reproductive benefits

Beside nutritional motivation, other benefits of cannibalism could have also promoted clutch cannibalism in female poison frogs. In species where adults are able to recognize their own offspring, selective cannibalism of unrelated clutches can be a powerful mechanism to prevent other individuals from successful reproduction as well as to monopolize mating events within a population. If parental investment in single clutches is reduced with increasing number of simultaneously present clutches (cf. [68]), cannibalism of unrelated clutches could function to secure the male’s paternal effort for the females’ own clutches (Parental quality hypothesis, [46]). Such a mechanism has previously been suggested for *Dendrobates auratus*, [48], where females actively compete for access to males and intensity of parental care is expected to decrease with increasing number of clutches of a given male. However, in *A. femoralis* females do not compete for access to male mating partners and also the quality of parental care does not seem to be dramatically affected by increasing clutch numbers of a single male [56–58, 60, 62]. Male *A. femoralis* were observed guarding up to five clutches simultaneously [58] and parental care is limited to tadpole transport, which might not require extended time investment by the father. Thus, we do not assume that females cannibalize foreign clutches to increase the amount of parental care provided to their own clutches or other reproductive benefits.

In some animal species, the killing of conspecific young is a sexually selected mechanism that mainly serves to make a prospective mating partner that is currently parenting become receptive sooner. Such behaviour has been documented in various taxa including mammals [16] and birds [69]. A similar mechanism was even suggested for anecdotal observations of clutch cannibalism by male *Oophaga pumilio* ([50], see also [70]). However, *A. femoralis* females have never been observed to fight for males or mating opportunities. Furthermore, males were observed to care for up to five clutches simultaneously [58] and also do not specifically manipulate or actively defend their clutches (pers. obs. E. Ringler, this study). In our study, we only included males that had previously sired only one single clutch, in order to provide standardized conditions across trials. Thus, we cannot make inferences about whether paternity quality is reduced with increasing number of clutches, and if clutch cannibalism by females is in turn a meaningful measure to ensure minimum levels of paternal care. However, we speculate that increasing number of clutches (at least in the range of naturally observed maximum numbers) does not reduce the respective parental care each single clutch receives to a relevant degree, and also does not limit the number of mating opportunities, as males are able to attend several clutches at a time. Therefore, we think that ensuring a certain amount of parental care by the father might not be a primary motivation for cannibalizing foreign clutches by *A. femoralis* females.

### Predation risk and competition

In situations where cannibalism reduces the future tadpole population size and therefore the number of intraspecific competitors, cannibalizing foreign clutches may enhance the survival of one’s own offspring. In species where tadpoles prey on other tadpoles, as it is the case for example in *D. auratus*, cannibalizing other females’ clutches could reduce the level of cannibalism towards their own offspring if tadpoles from multiple clutches end up in the same water body [71]. Tadpoles of *A. femoralis* are not cannibalistic (pers. obs. E. Ringler) and are usually distributed across different pools of water by males [72, 73]. Females would therefore have to exhibit extremely high rates of cannibalism – which however has not been observed in natural populations of *A. femoralis* – to considerably reduce future tadpole competition for their own offspring. We consequentially doubt that the motivation of cannibalistic female *A. femoralis* is to reduce the risk of predation by other tadpoles.

### Factors reducing clutch cannibalism

In our study, cannibalism was generally higher in conditions where no male was present at any time during the experiment, suggesting a prominent role of male presence for inhibiting clutch cannibalism by female poison frogs. In *A. femoralis*, clutches are laid inside male territories that are heavily defended against conspecifics [54, 55]. In other amphibians, egg-guarding by parents has been shown to be a powerful strategy to reduce predation risk [32]. For example, egg predation in *E. coqui* is in great measure caused by conspecifics, therefore males actively defend their nests to reduce heterocannibalism [35]. Males of Fornasini’s spiny reed frog, *Afrixalus fornasini,* construct folded-leaf nests, which can also be seen as a form of parental care to prevent clutch cannibalism by other adult frogs, as both males and females cannibalize eggs and developing larvae [74].

In *A. femoralis*, active protection of clutches by fathers was never observed, neither in any previous nor in this study. Male presence inside the tank was enough to prevent females from approaching and preying on unrelated clutches. Consequentially, we suggest that the high level of territoriality in *A. femoralis* males might simultaneously serve to protect clutches against cannibalistic conspecifics – males and females. A similar mechanism was proposed for *Cophixalus parkeri*, where males defend their nest sites and thereby possibly reduce clutch cannibalism by other individuals [75]. These findings suggest that male territoriality might be an important prerequisite for, or by-product of the evolution of male parental care, and that active defence of clutches may be interpreted as a more derived form of territorial defence behaviour.

In this study, clutch cannibalism by females was generally lower in the presence of a male and preceding oviposition. We were not able to test for the effects of male presence alone, as when a female was in a terrarium with a male they immediately produced a clutch together. When males were completely absent, and females had no clutch of their own, levels of cannibalism were higher than in a terrarium with a male and a clutch of their own. When females had produced their own clutch and the male was removed afterwards, cannibalism rates were intermediate to those of the conditions with males either entirely present or absent. Interestingly, the two females that cannibalized the foreign clutch when a male was present were also the only two out of ten from this trial that had not produced their own clutch previously, highlighting the prominent role of oviposition in mediating aggressive and affiliative behaviours in *A. femoralis* females. Similar observations typically are known from studies in mammals, where females generally show aggressive behaviour towards unrelated pups, but switch into affiliative behaviour soon after mating [76–78]. For example, female house mice, *Mus domesticus*, generally kill unrelated pups, but when they are lactating, they do not attack unrelated pups that are of similar age to their own offspring, and even adopt new-born pups [77]. Also, in Mongolian gerbils, *Meriones unguiculatus*, unmated females cannibalize foreign pups, but in turn provide maternal behaviour towards foreign offspring during late pregnancy [78]. A similar relation between cannibalism and parenting was also previously found in frogs; adult *Cophixalus parkeri* cannibalize clutches only when they are not breeding; thereby reducing the risk of accidental filial cannibalism [75].

Considering the distant phylogenetic relationship of mammals and frogs and their different forms of parental care and mating systems, it is interesting that similar behavioural responses are observed in both groups during pregnancy, oviposition, or raising of their own young. Highly conserved hormonal and/or neuronal networks might be responsible for regulating parental and cannibalistic behaviour across all vertebrates [79, 80]. In mammals, the neuroendocrinological mechanisms underlying parental behaviour are relatively well investigated. Oxytocin and prolactin have been shown to be important regulators of affiliative behaviours, including parental care [81]. In contrast, the neuroendocrinal regulation of parental behaviour in amphibians is still poorly understood [82, 83]. The few studies on hormonal regulation of aggressive and parental behaviour in anurans provide rather inconclusive results [84]. In our study, the finding that cannibalism was reduced after oviposition in *A. femoralis* females suggests hormonal changes in females after mating, which might prevent females from being cannibalistic in the presence of their own clutches. The observed reduction in cannibalistic behaviour in the presence of a male, as discussed above, might likewise be linked to hormonal changes in females. When confronted with a courting male, females might immediately go into reproductive mode, where affiliative behaviours are promoted and any aggressive behaviour, including clutch cannibalism, is suppressed. Future studies should identify the distinct hormonal and neuronal mechanisms underlying parental decision-making in *A. femoralis*, which will also provide valuable insight into the evolution of parental behaviours across vertebrates.

## Conclusions

In summary, our study clearly demonstrates selective clutch cannibalism by *A. femoralis* females. Females might gain valuable nutrients from eating conspecific eggs, and possibly increase their inclusive fitness by selectively preying on unrelated clutches. Both male presence and oviposition reduced the extent and frequency of clutch cannibalism in female *A. femoralis*, which might be mediated via neuroendocrinological mechanisms associated with their own reproductive behaviour. The father’s simple physical presence reducing clutch cannibalism by females highlights the important role of female aggression and male territoriality for the evolution of male parental care.

## Additional files

Additional file 2: **Dataset S1.** (XLSX 12 kb)
